# Medulloblastoma Presenting As Isolated Leptomeningeal Enhancement With No Primary Mass

**DOI:** 10.7759/cureus.26598

**Published:** 2022-07-05

**Authors:** Molly Meister, Julian J Lin, Sarah E Bach, Yamini Kapileshwarkar, Prerna Kumar

**Affiliations:** 1 Medical School, University of Illinois College of Medicine Peoria, Peoria, USA; 2 Neurosurgery, University of Illinois College of Medicine Peoria, Peoria, USA; 3 Pathology, University of Illinois, Peoria, USA; 4 Pediatrics, Loma Linda Children's Hospital, Loma Linda, USA; 5 Pediatrics, University of Illinois College of Medicine Peoria, Peoria, USA

**Keywords:** medulloblastoma with no primary mass, large cell/anaplastic variant, persistently increased intra-cranial pressures, refractory seizures, sugar coating of meninges, leptomeningeal enhancement

## Abstract

Medulloblastoma presenting with diffuse leptomeningeal enhancement and no identified intra-parenchymal primary mass is extremely rare. A 14-year-old previously healthy boy presented with a three-week history of symptoms consistent with increased intracranial pressure (ICP). Magnetic resonance imaging (MRI) revealed diffuse leptomeningeal enhancement which prompted consideration of infectious, inflammatory, and neoplastic etiologies. The patient became rapidly unstable requiring the placement of an external ventricular drain (EVD) and induction of a phenobarbital coma for refractory seizures. The “sugar-coated” appearance of the abnormal enhancement and thickened tissues raised concern specifically for malignancy. The patient remained extremely unstable and ultimately required surgical decompression for increased ICP at which time a biopsy was obtained. Despite attempting bridging intra-ventricular chemotherapy, the patient, unfortunately, passed away, just 14 days from the initial presentation. Final pathology later confirmed the diagnosis of medulloblastoma. Awareness of medulloblastoma in the differential of diffuse leptomeningeal enhancement is crucial for early identification and treatment of this rare presentation. This case is the first pediatric report of primary leptomeningeal medulloblastoma without a primary mass involving the large cell/anaplastic variant.

## Introduction

Medulloblastoma is a neuroepithelial malignancy of primitive neuroectodermal origin and is the most common malignant pediatric brain tumor [1,2]. It accounts for 10% of all primary central nervous system (CNS) tumors in children less than 19 years old, though the peak incidence is in children between five and nine years old [1,2]. Medulloblastomas are tumors of the posterior fossa and classically present with symptoms associated with increased intracranial pressure (ICP) such as headaches and persistent vomiting. Medulloblastomas classically appear as well-defined masses in the cerebellum that enhance both computed tomography (CT) of the head and brain magnetic resonance imaging (MRI) with gadolinium contrast. In the setting of a primary mass, dissemination into the subarachnoid space, which can appear as diffuse leptomeningeal enhancement, is present in up to one-third of cases at diagnosis and constitutes CNS metastatic disease [1,3].

While most cases present with a primary mass in the cerebellum or vermis with occasional dissemination into the subarachnoid space, there have been extremely rare cases presenting with leptomeningeal enhancement alone and no identified intra-parenchymal primary mass. Ferrara described the first documented case as “primary leptomeningeal medulloblastoma” [4]. Early and accurate diagnosis in these cases poses a great challenge due to the rarity of this presentation. Additionally, since the typical differential diagnosis for isolated leptomeningeal enhancement in pediatrics includes various infectious or inflammatory processes (such as viral or autoimmune encephalitis, meningitis, neurosyphilis, and leptomeningeal carcinomatosis), medulloblastoma is often not even included in the differential diagnosis.

Due to advances in molecular analysis, there are four defined subgroups of medulloblastoma with distinct molecular markers and varying prognoses for long-term survival. Current treatment regimens are based on subgroup and risk classification and typically include surgical resection of the primary mass in conjunction with radiation and chemotherapy. “Primary leptomeningeal medulloblastoma” is particularly challenging to manage not only because it is difficult to diagnose, but also because gross total resection is not an option, optimal treatment remains poorly defined, and disease progression is rapid with patient survival ranging from weeks to months from diagnosis [2,3,5]. Early diagnosis and detection remain a significant barrier to treatment initiation and potential successful outcomes, especially due to the rarity of the presentation and broad differential for the initial presentation. Awareness of the possibility of primary leptomeningeal medulloblastoma is crucial for the timely management of these patients.

## Case presentation

**Figure 1:**
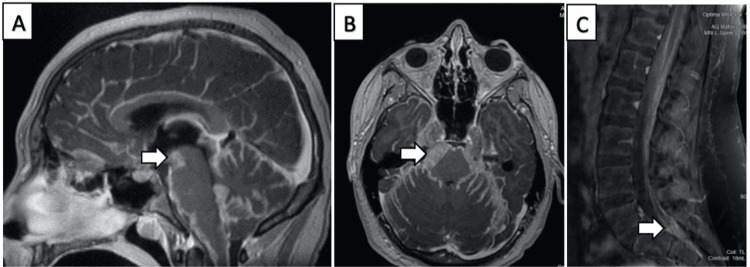
Brain MRI (A) Mid-sagittal T1 MRI of the brain with contrast enhancement demonstrates extensive “sugar coating” of the cerebral surface especially the cerebellum and brainstem.  Arrow points to bulky disease within the ambient cistern. (B) Corresponding axial T1 MRI of the brain with contrast showing extensive disseminated tumor surrounding the pons (arrow) and cerebellum. (C) Mid-sagittal T1 MRI of the lumbar spine with contrast showing “drop” metastasis at the end of the thecal sac (arrow) which was explored during surgery.

A 14-year-old male presented to the emergency room with a three-week history of double vision, headaches, right-sided facial numbness, dysarthria, dizziness, and associated gait difficulties. An initial physical exam revealed an inability to adduct the right eye and a slight tongue deviation to the right. He was also noted to have had a 17-pound weight loss in the preceding months. Sedated brain MRI with and without contrast revealed extensive diffuse leptomeningeal enhancement, evidence of vasculitis, and secondary hydrocephalus with crowding of the posterior fossa (Figures 1A, 1B).

The findings were most concerning for meningitis with additional possible differential diagnoses including leptomeningeal carcinomatosis, lymphoma, viral or autoimmune encephalitis, tuberculosis, or fungal infection. Given the hydrocephalus and neurologic deficits, the patient was transferred to the intensive care unit, electively intubated, and underwent an urgent EVD placement. Initial ICP was >20 cm H_2_O, which decreased to 8-13 cm H_2_O after drainage through the EVD. EVD output became bloody two hours after placement and CT revealed a new right intra-parenchymal hemorrhage along the drain tract. Cerebrospinal fluid (CSF) cultures and gram stain were negative. Flow cytometry was negative for malignancy. CSF cytology demonstrated a few atypical and primitive-looking cells suspicious for a neoplastic lesion but indeterminate for malignancy. On hospital day 3, a complete MRI of the spine was performed which revealed diffuse leptomeningeal enhancement concerning for leptomeningeal carcinomatosis, infectious etiologies, or inflammatory processes (Figure 1C). Neurosurgery felt a neoplastic process was very likely given the "sugar-coated" appearance of the notably thickened tissues.

On hospital day 4, the patient developed refractory seizures that required a pentobarbital coma along with other anti-epileptic drugs. He concurrently developed significantly elevated ICP that required aggressive medical management. At this time, he had no brainstem reflexes except dilated pupils that were only sluggishly reactive. The patient remained too clinically unstable for biopsy at this time. Exhaustive workup for typical and atypical organisms and various non-infectious conditions from both serum and CSF were not supportive of infectious or inflammatory processes and thus, anti-infective therapies were de-escalated. He developed acute respiratory distress syndrome and methicillin-sensitive staphylococcus aureus pneumonia on hospital day six requiring escalation of ventilator settings and the need for vasopressor support and antibiotics. The role of positron emission tomography (PET) scan was discussed to potentially identify a primary mass and to possibly find a more accessible biopsy site, but the patient remained too clinically unstable to undergo prolonged imaging studies.

On hospital day 8, the patient underwent a sub-occipital craniectomy for posterior fossa decompression at which time an S1 laminectomy was also performed for tumor biopsy. Bone marrow aspirate and biopsy were also performed. Bone marrow studies were negative for malignancy. Preliminary results from the tumor biopsy revealed a small blue cell tumor that was most likely a medulloblastoma. Pentobarbital infusion was able to be weaned and discontinued but unfortunately, seizures soon recurred and elevated ICPs again became an issue. Attempts to control the patient’s ongoing seizures and increased ICPs were largely unsuccessful, and he was too unstable to undergo emergent therapeutic radiation therapy. Intra-ventricular cytarabine was given on hospital days 10 through 12 in an attempt to control the disease. His family became increasingly concerned with the patient’s suffering given his rapid decline and uncertain neurologic prognosis. His parents made the decision to redirect goals of care to comfort care. The patient was extubated and peacefully passed away 14 days after the initial presentation.

**Figure 2:**
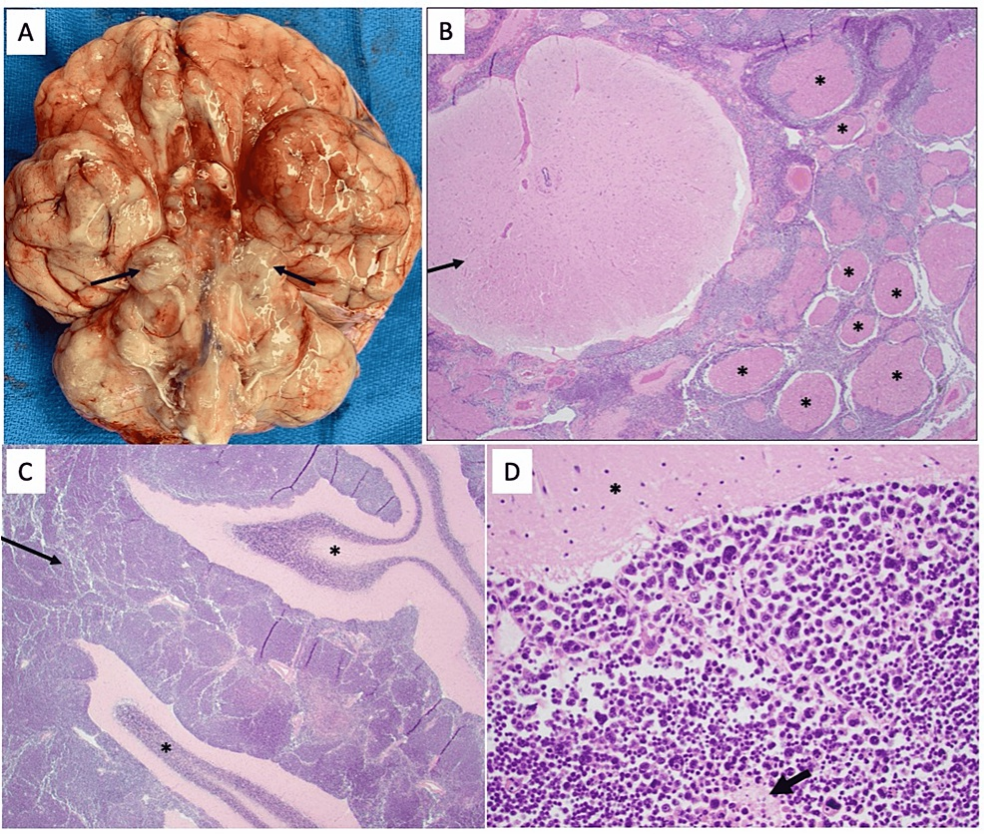
Gross and microscopic examination of tissue specimens (A) Gross examination of the fresh brain at autopsy revealed extensive leptomeningeal tumor infiltration over the base of the brain, especially striking over the surface of the brainstem and cerebellum, with encasement of cranial nerves and vessels. Nodular tumor deposition was also noted in bilateral cerebellopontine angles (arrows). (B) Microscopic examination of the spinal cord and cauda equina nerve roots revealed extensive circumferential encasement of the cord (arrow) and individual nerve roots (asterisks) by a small round blue cell tumor (H&E; 20x). (C) Microscopic examination of the cerebellum (asterisks) revealed extensive leptomeningeal infiltration by a small round blue cell tumor (arrow) (H&E; 20x). (D) Higher power examination of the leptomeningeal tumor reveals diffuse sheets of tumor cells with hyperchromatic ovoid to irregular nuclei, marked nuclear pleomorphism, apoptosis, and areas of necrosis (arrow), consistent with large cell/anaplastic morphology. The adjacent cerebellar cortex can be seen at the top (asterisk) (H&E; 200x).

Final pathology from the biopsy later revealed medulloblastoma, large cell/anaplastic variant, WHO grade 4, and non-WNT/non-SHH subtype (Figures 2A-2D).

Additional molecular analysis confirmed a p53 wildtype pattern, positive isochrome 17q, MYC amplification, and no MYCN amplification. The findings were consistent with Group 3 medulloblastoma.

## Discussion

Medulloblastoma with diffuse leptomeningeal enhancement and no primary tumor is extremely rare as only seven other cases have been reported [2-8]. Of these, approximately half have been in pediatric patients under the age of 18 with the remaining cases seen in adults. Patients presented with headaches, decreased consciousness, and recurrent vomiting. Additional reported symptoms included dysmetria, blurred vision, tinnitus, neck pain, ataxia, hearing loss, and seizures. Sub-occipital craniotomies and biopsies were performed in all cases, which revealed medulloblastoma, most commonly the classic variant of medulloblastoma. Six of the seven reported cases had poor prognoses, with death occurring days, weeks, and months following diagnosis. Treatment according to the Head Start III protocol was attempted in one of these cases, as well as radiation in another, with no disease response. The seventh patient presented in 2019 and is the only report involving the desmoplastic/nodular variant of medulloblastoma, which has a better prognosis in general [7]. The patient received induction chemotherapy with carboplatin, craniospinal irradiation, and radioimmunotherapy with ^131^I-burtomab. The disease burden resolved completely within one month and the patient had no evidence of recurrent disease radiologically at 17 months [7]. Only one prior case has been reported of primary leptomeningeal medulloblastoma involving the large cell variant and this was in an adult [8]. This presentation of medulloblastoma typically progresses rapidly with historically poor outcomes. Nonetheless, early diagnosis remains critical as it may allow for treatment sooner. Additionally, histopathology results that provide variant and subtype information may be helpful and prognostic; thus, early biopsy should be attempted if the patient is clinically stable. This case is the first reported pediatric patient with primary leptomeningeal medulloblastoma involving the large cell/anaplastic variant.

## Conclusions

Isolated diffuse leptomeningeal enhancement in the setting of increased ICP has a broad differential including infectious and inflammatory processes such as meningitis, autoimmune encephalitis, cerebral infarction, and meningeal carcinomatosis with a primary tumor outside of the CNS. Due to its rarity and its unique presentation with lack of a primary mass on imaging, primary leptomeningeal medulloblastoma is a challenging diagnosis to make. Primary leptomeningeal medulloblastoma should be considered in the differential diagnosis for presentations of diffuse leptomeningeal enhancement on MRI. This report describes a very rare presentation of medulloblastoma and is the first report of a pediatric patient with primary leptomeningeal medulloblastoma involving the large cell/anaplastic variant.
